# Effect of barley straw and Egyptian clover hay on the rumen fermentation and structure and fibrolytic activities of rumen bacteria in dromedary camel

**DOI:** 10.14202/vetworld.2022.35-45

**Published:** 2022-01-17

**Authors:** Alaa Emara Rabee

**Affiliations:** Department of Animal and Poultry Nutrition, Desert Research Center, Cairo, Egypt.

**Keywords:** *Camelus dromedaries*, cellulase and xylanase, forage type, hay and straw, rumen bacteria

## Abstract

**Background and Aim:**

Understanding the regulations of rumen microbiota and their fibrolytic capabilities under different forages are essential to improve rumen fermentation and animal feed efficiency. This study aimed to evaluate the changes in the rumen fermentation and the structure and fibrolytic activities of rumen bacteria in camels fed barley straw and Egyptian clover hay.

**Materials and Methods:**

Three fistulated camels were fed a diet containing barley straw for 30 days; then transitioned to a diet containing Egyptian clover hay for 30 days. In addition, bacterial media enriched with xylan and different cellulose sources, namely, filter paper, wheat straw, and alfalfa hay, were used to evaluate the ability of camel rumen bacteria to produce xylanase and cellulase enzymes.

**Results:**

The camel group fed Egyptian clover hay showed higher crude protein intake, rumen ammonia, total volatile fatty acids, and acetic acid. Moreover, the camel group fed barley straw showed higher neutral detergent fiber intake, rumen pH, and propionic and butyric acids. Principal component analysis showed that bacterial communities were separated based on the forage type. Forage type affected the composition of rumen bacteria and most of the bacterial community was assigned to phylum Bacteroidetes and Firmicutes. Egyptian clover hay diet increased the proportions of genus *Prevotella* and *Ruminococcus*; while fed barley straw diet increased the *Butyrivibrio*, *RC9_gut_group*, and *Fibrobacteres*. The bacterial culture of the Egyptian clover hay fed group produced the greatest xylanase and the bacterial culture of the barley straw fed group produced the maximum cellulase.

**Conclusion:**

Egyptian clover hay is recommended to feed camels in intensive production. Moreover, the bacterial community in the camel rumen is a promising source of lignocellulolytic enzymes.

## Introduction

Dromedary camel can withstand the adverse conditions of hot deserts due to its adaptability to heat stress, severe droughts [1], and it can utilize poor-quality fodder plants that are avoided by other herbivores [2]. Moreover, it can provide more meat and milk than other animals under harsh desert conditions [3]. Therefore, the camel is a key player animal in food security in drought regions [4]. Consequently, the camel farming system is changing from the pastoral system to the intensive system [5], and camel feeding is shifting from grazing in pastoral areas to concentrated supplements and high-quality forages in intensive farms [6,7]. Camel feeding and management for intensive meat and milk production need to be studied sufficiently to exploit the potential of the camel as a source of animal protein [7-10]. Dietary forage is important for rumen health and stability of the rumen ecosystem and affects the feed intake [11]. Farid *et al*. [9] indicated that camels fed different types of forage showed different average daily gains. Egyptian clover and straw of cereal crops are commonly used in animal feeding in Egypt [12]. Furthermore, the deficiency of high-quality forage such as alfalfa and Egyptian clover hay is the driving force to use low-quality forage such as crop straw in animal feeding [3,13].

The digestion of plant biomass in the rumen relies on the activities of symbiotic microorganisms, including bacteria, protozoa, fungi, and archaea, that convert feedstuff to volatile fatty acids (VFAs) and microbial protein that provide the host animal with energy and protein [14,15]. Rumen bacteria predominate the rumen microbiota and degrade a wide range of substrates, including protein, lipids, and a wide range of polysaccharides such as cellulose, hemicellulose, pectin, and starch [7,16]. Therefore, the abundance of rumen bacteria is primarily affected by the chemical composition of animal diet [17]. Subsequently, it is necessary to understand the changes in rumen fermentation and modulations of rumen bacteria under different feeding systems to create efficient and stable microbial communities, which optimally degrade ingested feeds and maximize animal productivity [18,19]. The rapid expansion of molecular techniques such as next-generation sequencing has enabled determining the changes in rumen microbiota under various treatment conditions, which offer the possibility to improve the digestibility of plant fiber and improve animal productivity [7]. A previous study suggested that the microbial community in camel can degrade poor-quality plant biomass [20-22]. Thus, the camel rumen microbiome could be a promising source of carbohydrate-active enzymes used in different biotechnological and industrial applications [21]. Some rumen bacteria were used to produce fibrolytic enzymes, including *Rumminococcus*, *Bacillus*, and *Clostridium* [23-25]. Therefore, camel rumen could be a promising enzyme source with commercial applications [21]. Cellulase and xylanase enzymes have a key role in feed additives manufacturing and the bioconversion of lignocellulosic biomass to animal feed or fermentable sugars for biofuel production [24,26-28]. Therefore, the demand for cheap and high active, and stable enzymes is growing rapidly [21,26].

Most of the studies conducted on rumen microbiota in dromedary camels [3,7,29] are surveys. Moreover, intensification of camel production is the main driver to study the effect of different diet types on performance and rumen fermentation to optimize animal productivity. Little information is available regarding the effect of forage type on the rumen fermentation and composition and fibrolytic activities of rumen bacteria in camel; Hinsu *et al*. [22] investigated the effect of diets differing in the forage source, *Pennisetum glaucum*, *Sorghum bicolor*, and *Zea mays*, on camel rumen bacteria. However, no studies determined the effect of Egyptian clover hay and barley straw on camel rumen bacteria and their fibrolytic activities.

This study aimed to evaluate the changes in the rumen fermentation as well as the structure and fibrolytic activities of rumen bacteria in camels fed barley straw and Egyptian clover hay. Furthermore, the ability of rumen bacteria to produce lignocellulolytic enzymes using different carbon sources was evaluated.

## Materials and Methods

### Ethical approval

The study was conducted under the guidelines of the Department of Animal and Poultry Production, Desert Research Center, Egypt. In addition, the study was approved by the Institutional Animal Care and Use Committee, Faculty of Veterinary Medicine, University of Sadat City (Approval number: VUSC00008). All methods were performed in compliance with the ARRIVE guidelines.

### Study period and location

The study was conducted from July to August 2019 (60 days) at Maryout Research Station, Desert Research Center, Alexandria, Egypt.

### Animals

Three fistulated camels with an average body weight of 455 kg were used in this study. Throughout the experiment, animals were offered two experimental diets different in forage type. Concentrate feed mixture (CFM) was offered at 1400 g/head and roughage was provided to all animals *ad libitum*. In the first experimental period, barley straw (*Hordeum vulgare*) was provided as sole roughage. Then, the animals were transitioned to the second experimental diet with the same concentrates mixture plus Egyptian clover hay (*Trifolium alexandrinum*) as sole roughage. Animals were fed every diet for 30 days before the sampling period. Feed intake was determined for every animal. Concentrate feed mixture consisted of corn 57.5%, soybean meal 23%, wheat bran 19%, limestone 2.5%, salt 1.5%, sodium bicarbonate 0.5%, premix 0.4%, and antitoxins 0.1%. The chemical analysis of barley straw, Egyptian clover hay, and CFMs are presented in Table-1.

**Table 1:** The chemical composition (%) of concentrates feed mixture, barley straw, and Egyptian clover hay.

Item	Dry matter	Ether extract	Crude protein	Neutral detergent fiber
Barely straw	88	3.1	4.75	67
Egyptian clover hay	89	2.65	13.03	50
Concentrate feed mixture	90	4.5	14.5	53

### Rumen samples and fermentation parameters

At the end of the adaptation period of 30 days, rumen contents were collected before feeding and strained by two layers of cheesecloth. The pH of rumen samples was recorded using a digital pH meter (WPA CD70, ADWA, Szeged, Hungary). The rumen liquor was used to analyze rumen ammonia-N (NH_3_-N) and total VFA, DNA isolation, and inoculation of rumen bacteria into cellulolytic and xylanolytic media to determine the production of cellulase and xylanase.

### Chemical analysis

Dry matter (DM) and crude protein (CP) of the experimental diets were analyzed according to the Association of Official Analytical Chemists (AOAC) [30]. Neutral detergent fiber (NDF) was determined according to Van Soest *et al*. [31] without sodium sulfite. Rumen ammonia and total VFA were determined according to the methods of Annison [30] and AOAC [32], respectively. In addition, individual VFAs were measured using high-performance liquid chromatography.

### Bacterial community analysis

#### DNA extraction, polymerase chain reaction (PCR) amplification, and sequencing

One milliliter of rumen sample was centrifuged at 14,000× *g*. The remaining pellets were used for DNA extraction by i-genomic Stool DNA Extraction Mini Kit (iNtRON Biotechnology, Inc., Korea) according to the manufacturer’s instructions. DNA was eluted in 50 μL elution buffer, and DNA quantity and quality were checked by agarose gel electrophoresis and nanodrop spectrophotometer (Thermo Fisher Scientific, Madison, Wisconsin, USA). The V4 region of the bacterial 16S ribosomal DNA gene was amplified using primers 515F and 926R [33]. PCR amplification was conducted under the following conditions: 94°C for 3 min; 35 cycles of 94°C for 45 s, 50°C for 60 s, and 72°C for 90 s; and 72°C for 10 min. PCR products purification and preparation for sequencing using Illumina MiSeq system were conducted according to the protocol described by Comeau *et al*. [34] in Integrated Microbiome Resource (Dalhousie University, Canada).

### Determination of copy number of bacterial 16S rRNA by quantitative PCR (qPCR)

Quantitative real-time PCR (qPCR) was used to determine the total bacterial 16S rRNA copy number in the rumen samples. Standards were generated using serial dilutions of DNA purified from *Prevotella* spp., *Ruminococcus albus*, and *Butyrivibrio hungatei* that were purchased from DSMZ (Braunschweig, Germany). Serial dilutions of the standards ranging from 10^1^ to 10^6^ copies of the 16S rRNA gene were used. The qPCR was performed using the Applied Biosystems StepOne system (Applied Biosystems, Foster City, USA). Bacterial primers F (5′-CGGCAACGAGCGCAACCC-3′) and R (5′-CCATTGTAGCACGTGTGTAGCC-3′) [35] were applied. The 10 μL reaction consisted of 1 μL genomic DNA, 1 μL of each primer, and 7 μL SYBER Green qPCR Master Mix (iNtRON Biotechnology, Inc.). The PCR conditions followed 40 cycles of 95°C for 15 s and 60°C for 60 s. The linear relationship between the threshold amplification (cycle threshold) and the logarithm of 16S rDNA copy numbers of the standards was used to calculate the copy numbers of rumen bacteria per μL of DNA.

### Bioinformatics analysis

All the paired-end Illumina raw sequence reads were processed in R (version 3.5.2) using the DADA2 (version 1.11.3) pipeline as described by Callahan *et al*. [36]. First, quality checks were conducted; clean reads were denoised, dereplicated, and filtered for chimeras to generate amplicon sequence variants (ASVs). Taxonomic assignment of sequence variants was performed using a combination of the functions assign Taxonomy and assignSpecies and was compared using the SILVA reference database.

### Cultivation of anaerobic rumen bacteria

The growth medium was the modification of medium 10 [37]. The composition of the growth medium was as follows (per 1000 mL distilled water): 2 g trypticase, 0.5 g yeast extract, 37 mL solution of K_2_HPO_4_·3H_2_O (0.6 g in 100 mL distilled H_2_O), 37 mL salt solution [0.16 g CaCl_2_·2H_2_O, 0.6 g KH_2_PO_4_, 1.2 g NaCl, 0.6 g (NH_4_)_2_SO_4_, 0.25 g MgSO_4_·7H_2_O in 100 mL distilled H_2_O], 1 mL Hemin solution (1 g L^−1^), 1 mL Resazurin solution (1 g L^−1^), 50 mL solution of Na_2_CO_3_ (8 g in 100 distilled H_2_O), 1 g L-cysteine HCl, 200 mL clarified rumen fluid, 1 mL vitamin mix, and 1 mL trace mineral solution that was described by McSweeney *et al*. [38]. Rumen fluid was clarified and an anaerobic medium was prepared according to McSweeney *et al*. [38]. To determine the xylanolytic activities of camel rumen bacteria, the growth medium was supplemented with birchwood xylan (100 mg/bottle) (X). To determine the cellulolytic activities, the growth media were supplemented with one of the three fiber sources, filter paper (FP) (2 disks/bottle), wheat straw (WS) (100 mg/bottle), and alfalfa hay (AH) (100 mg/bottle). The medium pH was adjusted at 6.8 and about 50 mL of media was tubed into 120 mL serum bottles under the steam of CO_2_; then, the bottles were sealed and autoclaved at 121°C for 15 min. Strained rumen samples from each animal were kept under the stream of CO_2_; then, 1 m of every rumen sample was inoculated into every serum bottle and two bottles were prepared for every sample for four media (X, FP, WS, and AH). Rumen bacteria were grown anaerobically at 39°C for 2 days.

### Cellulase and xylanase enzyme assay

Samples of growing bacteria were collected and centrifuged at 13,000× *g*, 15 min, 4°C and the supernatant was used for enzyme assays. Cellulase and xylanase activities (mU/mL) were measured using EnzChek cellulase substrate (Invitrogen, UK) that determines endo-1,4-β-glucanase and EnzChek Ultra Xylanase Assay Kit (Invitrogen) that determines endo-1,4-β-xylanase, according to the manufacturer recommendations.

### Statistical analysis

The statistical analyses were conducted using the IBM Statistical Package for the Social Sciences (SPSS) version 20 software (IBM Corp., NY, USA) [39] and PAST [40]. The differences in feed intake, rumen fermentation parameters, bacterial copy number, microbial diversity, the relative abundance of bacterial phyla and genera, and cellulase and xylanase productions were performed using unpaired t-test. Principal component analysis was performed using the data of the relative abundance of dominant bacterial genera, the values of alpha diversity metrics, rumen fermentation parameters, and enzyme activities. All the sequences were deposited to the Sequence Read Archive, NCBI, under the accession number: PRJNA743427.

## Results

### Chemical composition

The chemical compositions on DM basis of CFM, barley straw, and Egyptian clover hay are shown in Table-1. The results revealed that CP content was higher in CFM (14.5%) and clover hay (13.03%) compared to barley straw (4.75%). In contrast, barley straw showed higher NDF (67%) compared to CFM (53%) and Egyptian clover hay (50%).

### Feed intake and rumen fermentation

The results revealed that all animals consumed all the offered CFM. The results of total and roughage feed intake expressed as g/kg^0.75^ (kilogram metabolic body weight) are shown in Table-2. The results revealed that DM intake (DMI) was similar between the experimental groups. Meanwhile, the forage type affected the CP intake (CPI) and NDF intake (NDFI) significantly (p<0.05). The camel group fed Egyptian clover hay showed significantly higher CPI; while the camel group fed barley straw showed higher NDFI.

**Table 2:** Total feed intake and roughage feed intake of camels fed different forage types.

Item	FS		FH		Overall mean	SEM	p-value
	
	Mean	SE	Mean	SE			
Forage feed intake g/kg^0.75^							
DMI	33	0.57	33.7	0.5	33.35	0.36	0.401
CPI	1.55	0.028	4.9	0.07	3.2	0.75	<0.0001
NDFI	22.2	0.36	18.8	0.3	20.5	0.8	0.002
Total feed intake g/kg^0.75^							
DMI	46.2	0.57	46.9	0.47	46.5	0.36	0.401
CPI	3.5	0.03	6.8	0.07	5.14	0.75	0.0001
NDFI	29.2	0.36	25.8	0.29	27.5	0.8	0.002

SE=Standard error, DMI=Dry matter intake, CPI=Crude protein intake, NDFI=Neutral detergent fiber intake, SEM=Standard error of the mean, FS=barley straw, FH=Egyptian clover hay

Table-3 illustrates the effect of forage type on the rumen pH, ammonia, total VFA, VFA fraction, and bacterial population. The results revealed that rumen pH was significantly higher (p<0.05) in the camel group who received barley straw; and the camel group who received Egyptian clover hay revealed higher ammonia (p<0.05) and total VFA (p<0.05). Furthermore, the results revealed that the camel group fed Egyptian clover hay had higher acetic acid concentration, while the group fed barley straw showed higher propionic and butyric acids concentrations without significant differences. Moreover, the camel group fed barley straw showed a higher rumen bacterial population (p<0.05).

**Table 3:** Rumen fermentation parameters and bacterial population in the rumen of camels fed different forage types.

Paramter	FS		FH		Overall mean	SEM	p-value
	
	Mean	SE	Mean	SE			
PH	6.7	0.05	6.3	0.05	6.5	0.09	0.008
Ammonia, mg/dL	11.7	1.4	45.7	4.06	28.7	7.8	0.001
Volatile fatty acids, meq/dL	15.7	2.3	26.6	0.33	21.16	2.6	0.01
Acetic, %	63.5	0.7	68	3.5	65.75	1.9	0.328
Propionic, %	26.2	0.7	22.6	1.45	24.4	1.06	0.097
Butyric, %	10.3	0.3	9.3	2.3	9.8	1.07	0.693
Bacterial population*	7.6	0.1	6	0.28	6.8	0.38	0.006

* Bacterial population=Log10 copies/μL DNA. SE=Standard error, FS=barley straw, FH=Egyptian clover hay

### Microbial diversity

A total of 79,049 high-quality sequence reads were generated from Illumina MiSeq sequencing of V4 region on 16S rDNA in six camel rumen samples with an average of 13,175±1818 reads per sample (mean±standard error). Total sequence reads in the barley straw fed group were 34,302 with a mean of 11,434±1841; also, the total sequence reads in the Egyptian clover hay fed group were 44,747 with a mean 14,915±3180 sequence per sample. The number of ASVs and alpha diversity indices was higher in the group fed clover hay compared to the group fed barley straw without significant difference (Table-4).

**Table 4:** Alpha diversity metrics of microbial communities in the rumen of camels fed different forages.

Alpha diversity indices	FS		FH		Overall mean	SEM	p-value
	
	Mean	SE	Mean	SE			
Observed amplicon sequence variants	531.7	76.32	1049	214.3	790.3	154	0.085
Chao1	532.36	76.75	1051	214.7	791.7	154.4	0.085
Shannon	5.97	0.13	6.34	0.2	6.2	0.13	0.219
InvSimpson	328.24	44.91	402.7	59.3	365.5	37.2	0.374

SE=Standard error, SEM=Standard error of the mean, FS=barley straw, FH=Egyptian clover hay

### Analysis of bacterial community

The taxonomic analysis of the bacterial community in the rumen of camels under investigation revealed 12 bacterial phyla (Table-5). The bacterial community in the current study was dominated by phylum Bacteroidetes (70.4%) and Firmicutes (23.6%). Other phyla that made up more than 1% were Proteobacteria (1.6%), Spirochaetes (1.7%), and Tenericutes (1.5%). Bacterial phyla that represented <1% were Cyanobacteria (0.2%), Fibrobacteres (0.6%), and Planctomycetes (0.2%) (Table-5). Bacterial phyla observed only in the barley straw fed camel group were Verrucomicrobia, Kiritimatiellaeota, and Lentisphaerae. Moreover, the phylum Elusimicrobia was found only in the Egyptian clover hay fed group (Table-5).

**Table 5:** The relative abundances (%) of bacterial phyla in the rumen of camels fed different forages.

Phylum	FS		FH		Overall mean	SEM	p-value
	
	Mean	SE	Mean	SE			
Bacteroidetes	53.45	3.04	87.4	0.96	70.4	7.7	<0.0001
Cyanobacteria	0.17	0.003	0.26	0.045	0.2	0.03	0.129
Firmicutes	38.87	3.5	8.28	0.28	23.6	7.02	0.001
Fibrobacteres	0.7	0.2	0.48	0.15	0.6	0.13	0.439
Planctomycetes	0.25	0.06	0.15	0.03	0.2	0.03	0.196
Proteobacteria	2.5	0.44	0.64	0.32	1.6	0.5	0.026
Spirochaetes	1.4	0.45	2.1	0.5	1.7	0.35	0.382
Tenericutes	2.2	0.16	0.8	0.2	1.5	0.32	0.007
Verrucomicrobia	0.07	0	0	0	0	0	ND
Kiritimatiellaeota	0.22	0	0	0	0	0	ND
Lentisphaerae	0.12	0	0	0	0	0	ND
Elusimicrobia	0	0	0.09	0	0	0	ND

ND=Non-determined, SEM=Standard error of the mean, FS=barley straw, FH=Egyptian clover hay

Forage type affected the relative abundances of the bacterial phyla. Phylum Bacteroidetes was significantly higher (p<0.05) in the camel group fed Egyptian clover hay compared to the camel group fed barley straw. On the family level, the members of phylum Bacteroidetes were affiliated mainly to the family Prevotellaceae, Rikenellaceae, and unclassified Bacteroidales (Table-6). Family Prevotellaceae was predominated by genus *Prevotella* that was significantly higher (p<0.05) in the Egyptian clover hay fed group compared to the barley straw fed group. Member of family Rikenellaceae was assigned mainly to *RC9_gut_group* that was higher in the barley straw fed than the Egyptian clover hay fed group without significant difference. Unclassified Bacteroidales were significantly higher (p<0.05) in the Egyptian clover hay fed group than the barley straw fed group (Table-6). The relative abundance of phylum Firmicutes was significantly higher (p<0.05) in the barley straw fed group than the Egyptian clover hay fed group. The members of this phylum were assigned mainly to the family Lachnospiraceae, Ruminococcaceae, and Family_XIII. Family Lachnospiraceae was significantly prevalent (p<0.05) in the barley straw fed group than in the Egyptian clover hay fed group; this family was dominated by genus *Butyrivibrio* that followed the same trend. In addition, some genera were observed exclusively in a specific group, such as *Acetitomaculum* and *Moryella* that were found only in the Egyptian clover hay fed group; also, genus *Lachnoclostridium* was found only in the group, the barley straw fed group (Table-6).

**Table 6:** The relative abundances (%) of bacterial families and genera in the rumen of camels fed different forages.

Family and genus	FS		FH		Overall mean	SEM	p-value
	
	Mean	SE	Mean	SE			
Phylum: Bacteroidetes							
Family Prevotellaceae	26.9	3.5	48.8	1.5	37.9	5.2	0.005
*Prevotella*	22.3	3.98	39.9	1.9	31.1	4.4	0.017
Rikenellaceae_*RC9_gut_group*	15.3	1.8	11.5	0.17	13.4	1.17	0.166
Unclassified_ Bacteroidales	11.1	0.4	27.07	1.1	19.1	3.6	<0.0001
Phylum: Firmicutes, family: Lachnospiraceae							
Family Lachnospiraceae	5.3	1.2	1.2	0.39	3.3	1.07	0.032
*Butyrivibrio*	1.1	0.19	0.2	0.04	0.7	0.2	0.012
*Acetitomaculum*			0.35				ND
*Moryella*			0.35				ND
*Lachnoclostridium*	0.65						ND
Phylum: Firmicutes, Family_XIII							
*Anaerovorax*	1.3	0.2	0.17	0.02	0.7	0.26	0.041
*Mogibacterium*	0.08						
Phylum: Firmicutes, family: Ruminococcaceae							
Family Ruminococcaceae	16.9	2.1	5.3	0.5	11.1	2.8	0.006
*Papillibacter*	3.52	0.09	0.7	0.22	2.11	0.63	<0.0001
*Ruminococcus*	1.01	0.09	1.48	0.18	1.24	0.14	0.091
*Saccharofermentans*	2.06	0.11	0.27	0.08	1.17	0.4	<0.0001
Unclassified_Ruminococcaceae	3.3						ND
Phylum: Firmicutes, other families							
Christensenellaceae_R-7_group	5.5						ND
Lactobacillaceae_*Lactobacillus*			0.05				ND
Erysipelotrichaceae_UCG-004			1.45				ND
Acidaminococcaceae _*Succiniclasticum*	7.5						ND
Clostridiales_vadinBB60_group			6.25				ND
Phylum: Spirochaetes							
*Treponema*_2	0.66	0.25	1.04	0.44	0.85	0.24	0.503
*Sphaerochaeta*	0.9	0.2	0.95	0.17	0.93	0.12	0.873
Phylum: Tenericutes							
Anaeroplasma			0.49				ND

ND=Non-determined, SE=Standard error, FS=barley straw, FH=Egyptian clover hay

The relative abundance of family Ruminococcaceae was significantly higher (p<0.05) in the barley straw fed group than in the Egyptian clover hay fed group. In addition, the family members were assigned mainly to genus *Papillibacter* and *Saccharofermentans* that were higher in the barley straw fed group, and genus *Ruminococcus* that was higher in the Egyptian clover hay fed group. Uncultured_Ruminococcaceae was observed only in the barley straw fed group. Some families within phylum Firmicutes were observed in a specific group, including family Lactobacillaceae that was assigned to genus *Lactobacillus*, which was observed in the Egyptian clover hay fed group only. In addition, family Acidaminococcaceae that was affiliated to genus *Succiniclasticum* was observed only in the barley straw fed group. Phylum Spirochaetes was higher in the Egyptian clover hay fed group and was assigned to genus *Treponema* and *Sphaerochaeta* that were higher in the Egyptian clover hay fed group. The members of phylum Fibrobacteres, Planctomycetes, and Proteobacteria were higher in the barley straw fed group and members of phylum Cyanobacteria were higher in the Egyptian clover hay fed group.

### The production of lignocellulolytic enzymes

#### Xylanase production

The anaerobic bacteria in the rumen of dromedary camels under different forage types were tested for their ability to produce xylanase enzymes (*in vitro*) by incubating rumen samples in anaerobic bacterial media containing birchwood xylan for 48 h. It could be noticed that higher xylanase production was associated with rumen samples collected from camels fed Egyptian clover hay fed (388.7±58.3 mU/mL) than camels fed barley straw fed (165.7±6.6 mU/mL) with a significant difference (p<0.05).

#### Cellulase production

In this study, rumen samples were inoculated into bacterial media containing different cellulose sources, FP, WS, and AH, for 48 h. Cellulase production varied according to the camel group and cellulose source. Maximum cellulase production was obtained when rumen samples of the barley straw fed group were inoculated into a bacterial medium supplemented with AH (Table-7). Pillai’s trace multivariate and Tukey tests were used to assess the significance of differences in cellulase production between the camel groups (barley straw fed and Egyptian clover hay fed) and cellulose sources (FP, WS, and AH), and it was observed that the difference between the camel groups was non-significant. However, the effect of cellulose sources on cellulase production was significant (p<0.05) and the interaction between the camel group and cellulose source was significant (p<0.05). The t-independent test was used to examine the cellulase production between the camel groups using the cellulose sources separately and the results revealed that the differences in cellulase production between the camel groups were significant (p<0.05).

**Table 7:** Cellulase (endo-cellulase) activity (mU/mL) (mean±SD) of rumen bacterial community of the dromedary camel fed different forages using different cellulose sources.

Cellulose source	FS		FH		Overall mean	SEM	p-value
	
	Mean	SE	Mean	SE			
Filter paper	146.34	34.35	18.8	2.2	82.5	32.4	0.053
Wheat straw	219.9	113.9	59.6	18.7	139.8	62.8	0.017
Alfalfa hay	1090.1	210.05	305.5	7.8	697.8	199.02	0.001

SE=Standard error, SEM=Standard error of the mean, FS=barley straw, FH=Egyptian clover hay

#### Principal component analysis

**Figure-1:**
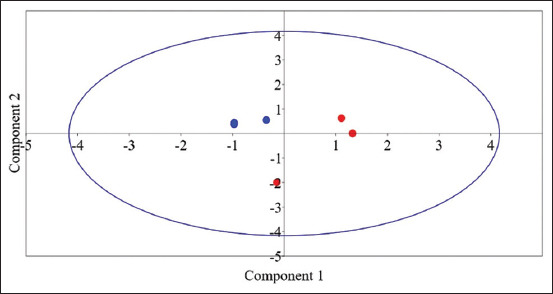
Principal component analysis of rumen bacterial community based on Bray-Curtis dissimilarity in the rumen of camels fed different forage types. The blue circles for camels fed barley straw and red triangle for camel fed clover hay.

Principal component analysis was conducted based on the value of rumen pH, ammonia, total VFA, enzymes productions, microbial diversity indices, and the relative abundances of bacterial groups. The result revealed that the samples were separated based on forage source (Figure-1).

## Discussion

Understanding the regulations of rumen microbiota and their fibrolytic activities under different feeding regimes is the cornerstone to improving rumen fermentation and animal productivity [26,41]. This study was conducted to evaluate the effect of forage type on the rumen fermentation, and composition and diversity of rumen bacteria and their cellulolytic and xylanolytic activities. Two types of forages were fed to the camels under investigation, barely straw and Egyptian clover hay. The Egyptian clover is commonly used in animal feeding in Egypt and is considered as a high nutritive value fodder regarding the protein and carbohydrates contents [7]. The chemical composition of Egyptian clover hay and CFM was in the ranges that were reported by Rabee *et al*. [3,12] (Table-1). Barley straw fed is abundant lignocellulosic biomass worldwide and it has high-fiber and low-protein contents [42]. The chemical composition of barley straw fed was in the range indicated by the previous studies [43,44]. Liu *et al*. [45] explained that higher NDF content in straw results in slow degradability in the rumen.

### Feed intake and rumen fermentation

Camels in this study offered free forage and the values of DMI were indicated in the previous studies on camels (Table-2) [46,47]. Forage type did not affect the DMI, which was supported by the previous studies on cattle [11,48]. In the same time, CPI and NDFI were affected by forage type, which agrees with Farid *et al*. [9]. Egyptian clover hay contains higher CP; while barley straw fed has higher NDF that explains the differences in CPI and NDFI. Lower rumen pH was linked to higher total VFA production in the Egyptian clover hay fed group that could be explained by the availability of soluble carbohydrates in clover hay compared to the barley straw [49-51]. The higher production of rumen ammonia in the Egyptian clover hay fed group that fed Egyptian clover hay is illustrated by higher CPI (Table-3) [52,53]. Wang *et al*. [48] compared the effect of different forage types on the feed intake, rumen fermentation in cows. They found that DMI was not affected and total VFA was higher in the group fed hay compared to the groups fed corn stover or rice straw that supports our findings.

The concentrations of acetic, propionic, and butyric acids in the current study were in the range observed by Dadvar *et al*. [54] for camels fed different forage plants. Camels fed barely straw showed lower acetic acid concentration (Table-3); a similar finding was obtained by Xu *et al*. [11]; and Zhang *et al*. [51] indicated that the inclusion of middle-quality forage such as *Leymus chinensis* in the animal diet increased the total VFA and acetic acid production.

### Rumen bacterial community

Forage type did not affect the microbial diversity significantly (Table-5); this finding was also indicated by Hinsu *et al*. [22] in camels fed different forages. The majority of rumen bacteria in the current study were affiliated to phylum Bacteroidetes and Firmicutes that were affected by forage type (Tables-6 and 7), which was also indicated by the previous studies on camel and cattle [3,7,22,51]. Most of the phylum Bacteroidetes was assigned to genus *Prevotella* that showed higher representation in animals fed Egyptian clover hay fed; a similar result was obtained by the previous studies [22,45,51] on cattle and camel fed different forages. Zhang *et al*. [51] reported that the prevalence of *Prevotella* in the rumen indicates the importance of this genus in rumen fermentation. This genus is involved in the degradation of different substrates in the rumen, including protein, xylan, pectin, and starch [7,45,51], illustrating the greater relative abundance in the Egyptian clover hay fed group that fed Egyptian clover hay that provides different growth substrates. Liu *et al*. [45] reported that *Prevotella* was correlated positively with protein content; therefore, it showed higher presentation with Egyptian clover hay fed group in the current study. Genus *RC9_gut_group* within phylum Bacteroidetes is highly specialized in lignocellulose degradation, which demonstrates the high representation in the barley straw fed group, fed low-quality forage [3,22,55].

Members of phylum Firmicutes were dominated by *Butyrivibrio*, *Papillibacter*, *Ruminococcus*, and *Saccharofermentans* that agree with the previous studies [45,56-58]. Genus *Butyrivibrio* and *Ruminococcus* are polysaccharide-degrading bacteria [45]. *Papillibacter* genus was previously found in a high proportion in cattle fed corn stover, which supports our results [51], and could indicate that this genus is involved in fiber digestion [59]. Furthermore, genus *Succiniclasticum* can degrade fiber and cellobiose [58], and was isolated from the rumen of animals fed grass silage and convert succinate to propionate [60], which might illustrate the presence of this genus in the barley straw fed group only and support the higher propionate in the barley straw fed group. *Saccharofermentans* is involved in polysaccharides degradation and produces acetate and propionate [61] explaining the higher proportion of this genus in the barley straw fed group.

Genus *Fibrobacteres* is specialized in cellulose degradation and was positively associated with higher NDF in animal diet [7,17,45,51], explaining its higher representation in the barley straw fed group that was fed barley straw fed. Phylum Spirochaetes was dominated by genus *Treponema* that was higher in the barley straw fed group. Rabee *et al*. [7] indicated that *Treponema* is fiber-associated bacteria and has potential fibrolytic activities, which ensure our findings. The results showed that the bacterial community was dominated by genus *Fibrobacteres*, *Prevotella*, *RC9_gut_group*, *Butyrivibrio*, *Papillibacter*, *Ruminococcus*, *Saccharofermentans*, *Sphaerochaeta*, and *Treponema*, which are consistent with the previous studies [45,56-58]. Furthermore, these findings indicated the importance of these genera in the utilization of forage in the rumen [45,57]. Liu *et al*. [45] studied the degradability and colonization of rice straw and hay by rumen bacteria in cows and concluded that physical structure and chemical characteristics are the main determiners of microbial colonization; also, forage type affected the relative abundance of colonizing bacterial genera, wherever *Prevotella* was higher in alfalfa; *Fibrobacteres* and unclassified Ruminococcaceae were higher in rice straw; these findings support our findings. A higher proportion of the bacterial community in this study was found unclassified, including unclassified *Bacteroidales* and unclassified Ruminococcaceae; and these bacteria were affected by the forage type [7,22,51]. Unclassified Bacteroidales were higher in the Egyptian clover hay fed group; while, unclassified Ruminococcaceae were found only in the barley straw fed group; a similar result was found by Zhang *et al*. [51]. Stiverson *et al*. [62] reported that unclassified bacteria might have a role in biohydrogenation in the rumen, which indicates that forage type affects the composition of fatty acids [51]. Liu *et al*. [45] indicated that unclassified bacteria were associated with NDF content indicating their role in degrading low-quality forage.

### Enzymes production

This study gets insights into the ability of anaerobic bacteria in the rumen of camels to produce xylanase and cellulase using birchwood xylan and different cellulose sources, FP, WS, and AH. Cellulase and xylanase production is inducible as it depends on various factors such as inoculum size, pH value, temperature, medium additives, fermentation time, and growth substrate [12,23,26,28,63]. Xylanase and cellulase productions were in the range indicated by similar studies [64-66] for rumen bacteria. On the other hand, the anaerobic bacterial community in this study produced more xylanase and cellulase than the aerobic fungi [67] and anaerobic rumen fungi of the camel gut [26]. Moreover, cellulase production was similar to the production of some commercial bacterial strains such as *Escherichia coli* [68] and cellulolytic bacterial consortium [69], highlighting the higher cellulolytic activities of rumen bacteria in camel. Cellulase production varied greatly between cellulose sources, and the highest cellulase production (1090 mU) was observed by anaerobic bacteria incubated in AH media supplemented by alfalfa hay, which is in agreement with similar studies [23,24,63]. The variation in cellulase production among the substrates in the current study could be attributed to the variation in the composition of the bacterial community associated with the carbon sources [29].

Animal diet affected xylanase and cellulase productions and higher xylanase production was observed by rumen bacteria of the Egyptian clover hay fed group. Higher cellulase production was observed with the bacterial culture of the barley straw fed group that was inoculated in AH media. The feeding system, including diet composition and feeding plan, is the primary determiner of the composition of rumen microbial communities [70]. For example, high-fiber diets stimulate the cellulolytic and hemicellulolytic microbes; while, starch and sugars are the major fermentation components of concentrate-based diets; thus, favoring the amylolytic microbes [71]. Our results indicated that forage type affected the composition of rumen bacteria. For example, the Egyptian clover hay fed group showed higher *Prevotella* that have xylanolytic activities [7,45] and *Ruminococcus* that have cellulolytic and xylanolytic activities [23,45]. These findings explain the higher xylanase production associated with the Egyptian clover hay fed group. Meanwhile, the camel group barley straw fed showed higher relative abundances of cellulolytic bacteria such as *Fibrobacter, RC9_gut_group, Butyrivibrio, Papillibacter, Saccharofermentans*, and *Treponema* [45,56-58] that explain higher cellulase production in the barley straw fed group. Samsudin *et al*. [29] inoculated rumen fluid from dromedary camels into anaerobic bacterial media enriched with three different fiber sources, including cotton thread, FP, and NDF from lucerne hay, and the results showed that the fiber type influenced the composition of bacterial community that grows in the fiber-enriched medium.

Moreover, members related to the phylum Firmicutes were dominant and some of the bacteria involved in the fiber digestion were assigned to *Fibrobacteres*. Thus, the higher cellulase and xylanase production associated with bacterial community of the Egyptian clover hay and barley straw fed camel groups could be attributed to the high proportion of fibrolytic bacteria in the original rumen content. This study highlights the rumen content of camels, is a promising source for lignocellulolytic enzymes and lignocellulolytic bacteria for commercial production of cellulase and xylanase enzymes.

The interaction between the anaerobic bacterial community leads to effective degradation of cellulose as the fiber degradation requires different types of cellulases and xylanases, which could not be produced together by many bacterial strains; therefore, it is challenging to utilize cellulose by pure bacterial cultures [72]. Thus, using mixed bacterial community in enzyme production is an effective technique to promote the utilization of poor-quality forages. The results of PCA explained that forage type affected chemical composition of animal diet and CPI and NDFI. Consequently, the rumen fermentation and composition of rumen bacteria were affected. Moreover, the cellulolytic and xylanolytic activities of rumen bacteria were affected.

Camel is well adapted to desert harsh conditions by unique grazing behavior and morphophysiology of the digestive system [73]. In addition, the retention time of ingested feed is longer in camel rumen than other ruminants, which improves the efficiency of plant biomass fermentation and that might explain the prevalence of fibrolytic and potential fibrolytic microorganisms in the camel rumen compared to other ruminants [7]. For instance, camel rumen contains higher relative abundances of *RC9_gut_group*, *Fibrobacteres*, and Ruminococcaceae than sheep and cattle [74,75], which highlight the camel rumen as a potential source of fibrolytic microorganisms or their enzymes for commercial applications.

## Conclusion

This study expands our knowledge regarding the effect of forage type on rumen fermentation, rumen microbiota, and metabolic capabilities of rumen bacteria. Egyptian clover hay increased rumen ammonia and total VFA and barely straw maintained higher rumen pH. The proportions of some polysaccharide-degrading bacteria were increased by including Egyptian clover in the camel diet. Egyptian clover hay is suitable forage for camel fed under intensive production; however, the barley straw could be suitable alternative. This study could be a base to design a feeding plan for camels under intensive production.

## Authors’ Contributions

AER: Designed the study, performed the experiments, analyzed the data, prepared figures and tables, wrote and reviewed drafts of the manuscript, and approved the final draft.
